# Stable and efficient immobilization of bi-enzymatic NADPH cofactor recycling system under consecutive microwave irradiation

**DOI:** 10.1371/journal.pone.0242564

**Published:** 2020-11-18

**Authors:** Rong Chen, Qiuhui Wei, Xin Wei, Yuheng Liu, Xiaomin Zhang, Xiabin Chen, Xiaopu Yin, Tian Xie

**Affiliations:** 1 Key Laboratory of Elemene Class Anti-cancer Chinese Medicine of Zhejiang Province, Engineering Laboratory of Development and Application of Traditional Chinese Medicine from Zhejiang Province, Holistic Integrative Pharmacy Institutes, School of Medicine, Hangzhou Normal University, Hangzhou, P. R. China; 2 Faculty of Preventive Medicine of Medical College, Hangzhou Normal University, Hangzhou, P. R. China; Xinyang Normal University, CHINA

## Abstract

One of the challenges in biocatalysis is the development of stable and efficient bi-enzymatic cascades for bio-redox reactions coupled to the recycling of soluble cofactors. Aldo-keto reductase (LEK) and glucose dehydrogenase (GDH) can be utilized as the NADPH recycling system for economic and efficient biocatalysis of (*R*)-4-chloro-3-hydroxybutanoate ((*R*)-CHBE), an important chiral pharmaceutical intermediate. The LEK and GDH was efficiently co-immobilized in mesocellular siliceous foams (MCFs) under microwave irradiation (CoLG-MIA). while they were also co-immobilized by entrapment in calcium alginate without MIA as control (CoLG-CA). The relative activity of CoLG-MIA was increased to 140% compared with that of free LEK. The CoLG-MIA exhibited a wider range of pH and temperature stabilities compared with other preparations. The thermal, storage and batch operational stabilities of microwave-assisted immobilized LEK-GDH were also improved. The NADPH recycling system exhibited the potential as the stable and efficient catalyst for the industrial preparation of (*R*)-CHBE.

## Introduction

The continuous regeneration of expensive cofactors in their active forms increases the cost efficiency of the biocatalysis process. Particular attention has been devoted to the in situ regeneration of redox cofactors such as NADPH or NADH, by varieties of techniques (e.g., chemical, photochemical, and enzymatic) [1, 2]. Enzyme-mediated redox-cofactor recycling system is preferred because of the high selectivity and efficiency in the industrial manufacture [3, 4]. In the system, the desired redox reaction by the main enzyme as well as the cofactor and cofactor-recycling reaction by the recycling dehydrogenase are coupled and performed simultaneously [5]. Moreover, the immobilization of the enzymes that were involved in the redox cascade allows facile separation of immobilized enzyme and substrate product, no residual enzyme in the product, as well as the easy purification and high quality of the product. Immobilization may also promote diffusion limitations that reduce the catalytic efficiency of the multiple enzyme cascade [6, 7].

There are two strategies of immobilization of redox-cofactor recycling system: the main enzyme and the recycling dehydrogenases may be either separately immobilized onto two different carriers or co-immobilized onto the same carrier. The latter strategy was preferred because the larger amounts of cofactor would be recycled in situ inside the same porous particle and be ready to be used, in the vicinity of the main dehydrogenase. It was reported that the immobilization of multi-enzyme systems on the same solid surface that was activated by different reactive groups under different pH conditions. Although reversible immobilization using ionic exchange preserved the activity of both enzymes, the cofactor recycling would only be effective for 19% of the immobilized Tt27-ADH2. For the remaining 81% of Tt27-ADH2, cofactor regeneration would not be as efficient because its recycling partner would not be surrounding it. The spatial distribution of enzymes could be optimized by altering the immobilization rate in the presence of imidazol. However, the whole immobilization process was complicated, time-consuming, laborious and not guaranteed [5]. Moreover, it is ideally that both the main enzyme and recycling dehydrogenase were covalently attached onto the support to avoid enzyme leaching during the operational processes.

Aldo-keto reductases (AKRs) have emerged as an important tool for the asymmetric synthesis of pharmaceutical intermediates, such as (*R*)-4-chloro-3-hydroxybutanoate (CHBE), etc. The biocatalysis based on AKR is promising due to its high efficiency, high enantioselectivity, mild reaction conditions and environmental friendliness. However, production cost, poor stabilities in the reaction system, and dependence of NAD(P)H remained as bottlenecks in the application of enzyme catalysis. In recent years, glucose dehydrogenases (GDHs) play important roles in the coenzyme regeneration system by providing the hydrogen protons to NAD(P)^+^, which is essential for continuous asymmetric reductions [6]. Therefore, the co-immobilization of AKR and GDH has attracted considerable interests, for reducing costs, improved activity and desired stability. Among several methods, immobilization under microwave irradiation has been proved an efficient method [8].

Microwave radiation can induce bipolar molecular vibration, and its vibration frequency tends to coordinate with the external magnetic field vibration, resulting in the activation of molecules, the enhancement of diffusion and shortening the reaction time. Microwave radiation is more likely to affect the strong polar substances, such as proteins and peptides. Therefore, microwave radiation was introduced into the enzyme immobilization process to change the reaction state and uniformity of diffusion [9–11].

In our previous study, the AKR from *Lodderomyces elongisporus* NRRL YB-4239 (LEK) was demonstrated as a useful catalyst for the synthesis of optically pure (*R*)-CHBE with a satisfactory *e*.*e*. value, molar conversion and high production rate [12]. Furthermore, in this study, the LEK and GDH were co-immobilized in mesocellular siliceous foams (MCFs) under microwave irradiation for shorter immobilization period and enhanced diffusion, while entrapment of LEK and GDH in calcium alginate gel capsules without MIA was also performed as a control. The results of catalytic properties of the CoLG-MIA showed the potential as the stable and efficient catalyst for the large-scale production of (*R*)-CHBE.

## Materials and methods

### Materials and strains

The *E*. *coli* BL21 (DE3) strains, introduced plasmid pET28a-*lek* and pET22b-*gdh* were constructed and stored in the authors’ laboratory as previously described, respectively [12, 13]. Ethyl 4-chloro-3-oxodutanoate (COBE) was purchased from Alfa Aesar (Shanghai, China). Pre-functionalized mesocellular siliceous foams (MCFs-NH_2_) were obtained from Dr. Feifei Chen as the gifts. All other chemicals were provided by Sinopharm Chemical Reagent (Shanghai, China) and were analytical grade. Microwave^™^ Synthesis System was purchased from Discover (MARS5, CEM, USA).

### Expression and purification of LEK and GDH

The single colony of *E*. *coli* BL21 (DE3) (pET28a-*lek* or pET22b-*gdh*) strain was picked up from an overnight Luria Broth (LB) agar plate and subcultured into 100 mL of LB medium supplemented with 50 μg/mL kanamycin in a 1000 mL shake flask. The culture was carried on a rotary shaker (180 rpm) at 37°C. Isopropyl-β-D-thiogalactoside (IPTG) was added to a final concentration of 0.1mM when O.D._600_ determined by the Shimadzu MV-2550 spectrophotometer reached at 0.60. Cultures were incubated at 25°C for 15 h to induce protein expression. Then bacterial samples were collected by centrifugation at 10, 000 g for 20 min, and lysed in the sample buffer (50 mM phosphate buffer, pH = 7.4, 200 mM sodium chloride). Then cells were disrupted by ultrasonic wave for 30 min (work 4s, stop 4s). After centrifugation, the supernatant was used to purify His-tagged enzymes using Ni-NTA agarose for metal affinity chromatography (Qiagen, Hilden, Germany). Elution with a linear gradient of 20–500 mM imidazole (in 50 mM phosphate buffer, pH = 7.4, 200 mM sodium chloride) was performed to obtain the His-tagged protein. Sodium dodecyl sulfate-polyacrylamide gel electrophoresis (SDS-PAGE) was carried out on a 12% running gel under constant 120 V and then stained with Coomassie Blue. The concentration of enzymes were determined by the Bradford method [13]. The purified LEK and GDH were freeze-dried by lyophilizer (Labconco, U.S.A) with AUTO mode(precooling to -40°C, then the vaccum pump starts working until the vaccum lower than 0.12 mbar) and stored in -80°C for further usage.

### Co-immobilization of LEK and GDH by MIA

**Scheme 1:**
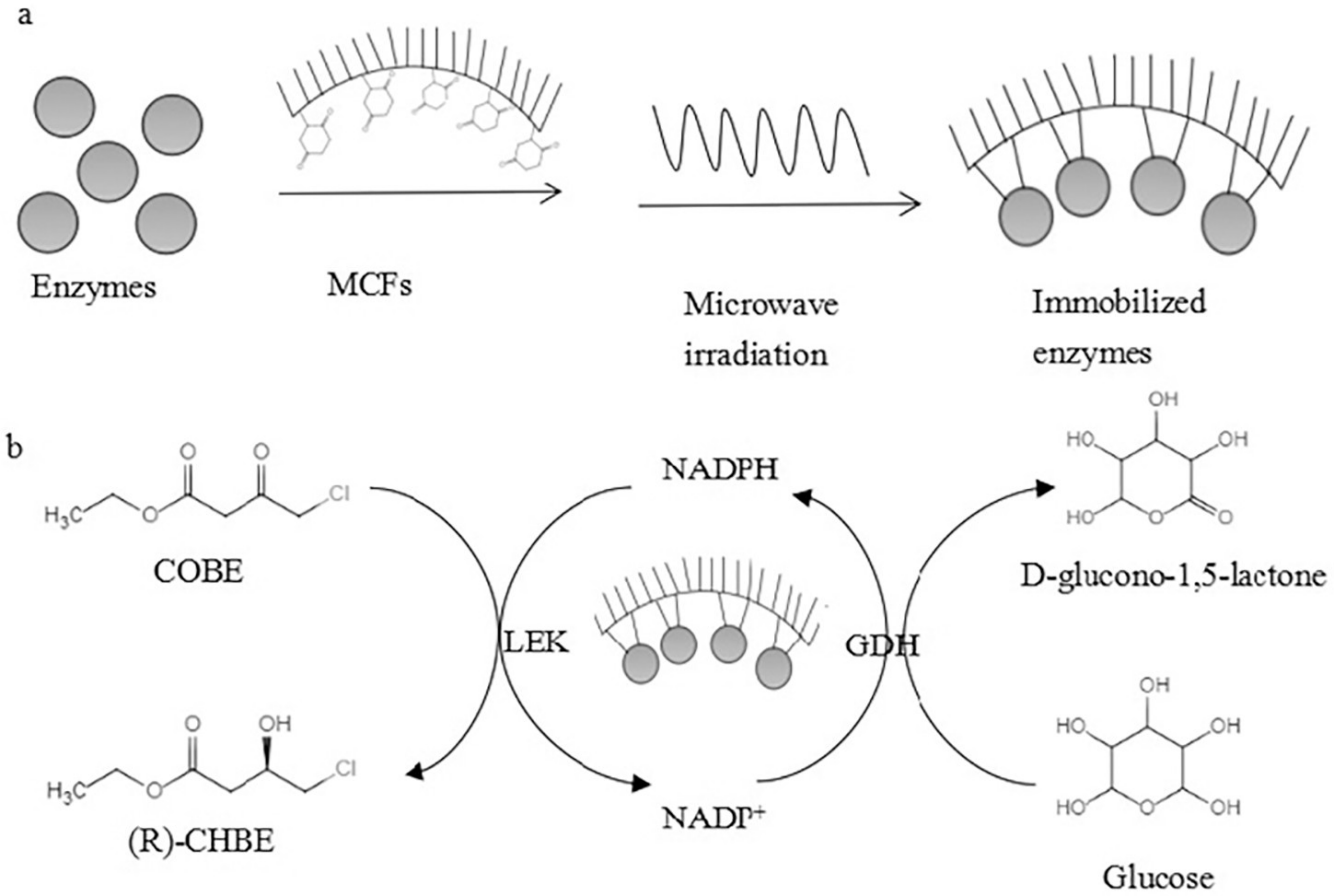
The strategies for immobilizing bi-enzymatic cascades for bio-redox reactions. a: The two enzymes, GDH and LEK were co-immobilized on the MCFs surface under microwave irradiation. b: The reduction of ketone (COBE) to afford a (*R*)-CHBE catalyzed by LEK and the enzymatic partner (GDH) as the recycling dehydrogenase to recycle the corresponding reduced cofactor (NADPH).

Immobilizing bi-enzymatic cascades for bio-redox reactions were shown in Scheme 1a. Firstly, 20 mg of pre-functionalized mesocellular siliceous foams (MCFs-NH_2_) were incubated in 3 ml of 0.1 M *p*-benzoquinone at room temperature for 2 h [14]. After washing with ethanol solution (20%, V/V) and distilled water, the MCFs-NH_2_ were suspended in 3 ml of 0.1 M phosphate buffer (pH 7.0) containing a total of 1.0 g purified enzymes. The molar ratio of LEK and GDH was 1: 1.5, calculated by their weight and molecular mass. Next, the immobilization was performed in an intelligent microwave reactor fitted with RPT-300 plus temperature control sensor (MARS5, CEM, USA). During the consecutive irradiation reaction process, drikold was added to maintain the certain temperature. The mixture was irradiated for a certain period in the microwave reactor. After centrifugation, the precipitation was washed twice with the sample buffer mentioned above. The effects of *p*-benzoquinone concentration, microwave power and irradiation time on the activity of co-immobilized enzymes were studied, respectively. Each assay was repeated at least three times.

### Co-immobilization of LEK and GDH by entrapment in calcium alginate gel capsules

Sodium alginate (SA) was dissolved in boiling water to a volume of 100 mL at five different final concentration (1, 2, 3, 4, 5%), respectively. The sodium alginate solutions were maintained at 30°C, mixed with enzymes and shook at 150 rpm for 30 min. The resulting mixture was extruded drop-wise through a 5 mm syringe into a CaCl_2_ solution with gentle stirring to form beads. Five different concentration of CaCl_2_ solution (0.5, 1.0, 2.0, 3.0, 4.0%) were tested. The gel capsules were kept in 4°C for 24 hours, washed by distilled water and dried by vacuum [14]. Hence, embedding of LEK and GDH in Ca-alginate gel capsules was obtained, namely CoLG-CA. The relative activities of CoLG-CA were tested under various concentrations of sodium alginate and CaC1_2_. Further, the effects of solidifying time on the activity of CoLG-CA were analyzed. Each assay was repeated at least three times.

### Enzyme activity assay

One unit of enzymatic activity was defined as the amount of immobilized enzymes required to catalyze the conversion of 1 μmol of COBE per minute. The assay mixture consisted of 25 mM potassium phosphate buffer (pH 7.0), 1.0 mM nicotinamide adenine dinucleotide phosphate (NADP), 15 mM COBE, 30 mM glucose (as the substrate of GDH for NADPH recycling), 2% (v/v) dimethyl sulfoxide (DMSO) and 1.0 g co-immobilization enzymes (Scheme 1b). The reaction was incubated at 37°C with shaking for 1 h, and then extracted twice with ethyl acetate. The yield of product was analyzed by Gas Chromatography (Shimadzu, Japan). Helium was used as the carrier gas at a constant flow rate of 3.0 mL/min. A GC column PEG-20M (0.25 mm i.d.×30 m, 0.25 μm film, Agilent, USA) was employed to separate the products using the following temperature program: ramp of 6°C min^-1^ from 90°C to 150°C followed by a 2 min hold, ramp of 10°C min^-1^ to 180°C for 15min. The injection port and detector temperature were 200°C and 230°C respectively [15].

The activities (A in the equation) of free LEK and GDH were determined spectrophotometrically according to the variation of absorbance values at 340 nm in one minute (D) [13, 15]. V means the volume of the reaction.

$$A=\frac{D\times V}{6.22}$$

The relative activity after immobilization was defined as the percentage of the activity of the immobilized LEK or free LEK. The activity assay was repeated at least three times.

### Effects of pH and temperature

To determine the optimum temperature for co-immobilized LEK-GDH, the enzyme activity was measured at 25°C to 50°C with the standard assay method described above. The effect of pH on enzyme was determined by comparing the relative activity of the enzyme incubated at various pHs ranging 5.5 to 9.0 at optimum temperature.

To evaluate the thermo-stabilities of these preparations, the immobilized enzymes were transferred into the optimal reaction medium and incubated at 25, 30, 35, 40, 45, 50°C for 30 min, respectively. Then samples of the suspension were withdrawn and used to assay the remaining activities as described above. For the pH-stabilities analysis, the immobilized enzymes were incubated in different buffer at pHs ranging 5.5 to 9.0 for 30 min. Then samples of the suspension were withdrawn, and then residual activities were assayed. The yield of product was analyzed by GC methods and deduced their relative activities. Each activity assay was repeated at least three times.

The effects of pH and temperature of free LEK and GDH were performed as previously described [12, 13].

### Storage and batch operational stability

For the study on storage stability, the co-immobilized preparations in solution were stored in sealed bottles at 4°C without further protection. Aliquots were taken periodically, and the enzyme activity was measured for three times. For batch operational stability tests, co-immobilized preparations were collected by centrifugation after enzyme activity assay, and then added in reaction system to analyze the residual activity, repeatedly.

### Statistical analysis

The values of data in the figures were the mean of three experiments. The error was less than 5%.

## Results and discussion

### Expression and purification of LEK and GDH

**Fig 1:**
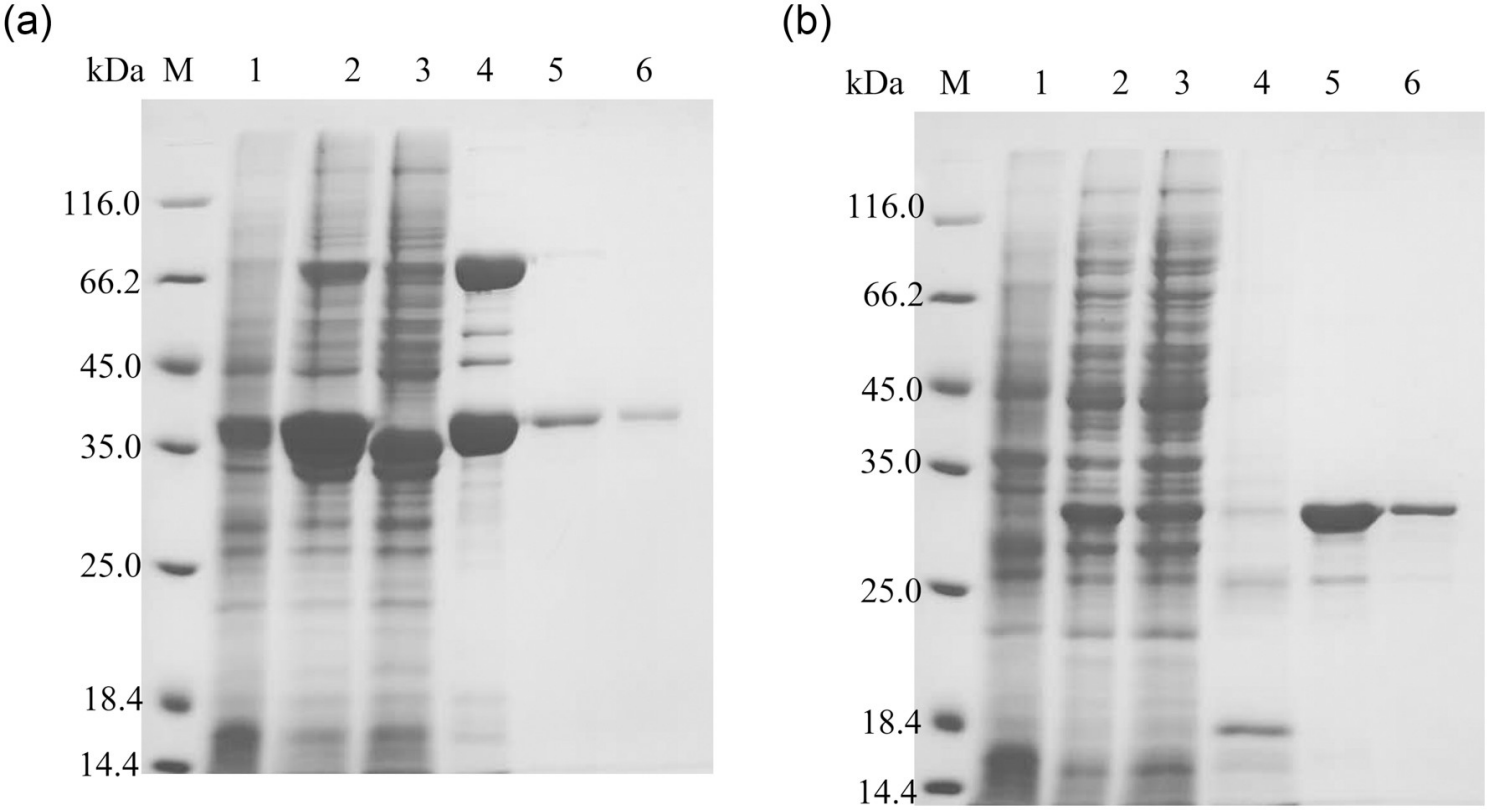
Expression and purification of His-tagged proteins. (a) LEK, (b) GDH. M: Protein molecular weight maker, Lane 1: The whole cell lysates before induction, Lane 2: The whole cell lysates after IPTG induction, Lane 3: Soluble fraction of the whole cell lysates after IPTG induction, Lane 4–6: Eluates collected with 100, 200 and 500 mM imidazole, respectively.

The *E*. *coli* BL21 (DE3) cells harboring the plasmid (pET28a-*lek*) or (pET22b-*gdh*104) were cultured in LB and added IPTG for inducing protein overexpression, respectively. The His-tagged protein was mainly released by the elution buffer of 200–400 mM imidazole, and the apparent purity was over 90% by gray scanning (Fig 1). The apparent size of the LEK was about 36.3 KDa. The concentration of purified LEK was 0.77 mg/ml (18.06 mg in total), isolated from 43.0 mg crude extracts as the starting material from 500 mL culture medium. The apparent size of the His-tagged GDH was about 29.6 KDa, in agreement with the calculated molecular weight. The concentration of purified GDH was 0.5 mg/ml, and the activity was 3.84 U/mg with glucose as the substrate. The purified LEK and GDH were freeze-dried and stored in -80°C for further use.

### Effect of the cross-linker content on CoLG-MIA

**Fig 2:**
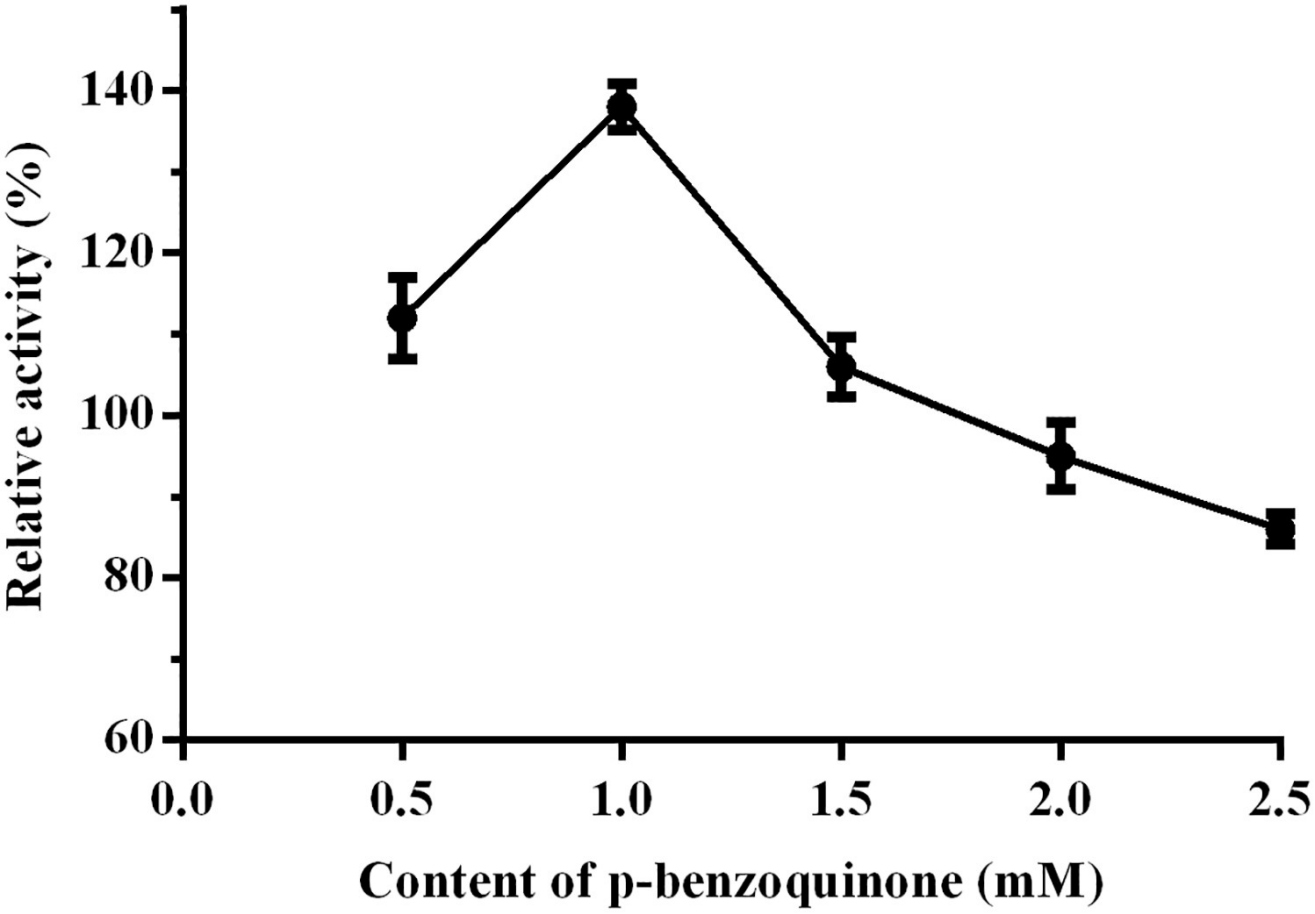
Effect of the *p*-benzoquinone content on the relative activity of LEK in the covalent immobilization.

In the previous work, *p*-benzoquinone was demonstrated to crosslink the nucleophilic groups of enzymes, with the amino groups on the walls of MCFs pores in form of covalent attachment [16, 17]. Because of the large surface areas and greater pore volumes, MCFs could enhance the loading capacity of enzymes and obtain high productivity and space-time yields [18]. MCFs is the most popular carrier for the uniform mesoporous diameter (2–40 nm) and large surface area (300–1500 M^2^/g), and preferred to be covalently attached. The covalent bonds can be formed between *p*-benzoquinone (as the linker in immobilization) and the nucleophilic group of enzymes or pre-functionalized mesocellular siliceous foams (MCFs-NH_2_). Therefore, LEK and GDH was co-immobilized in the pores of a functionalized MCFs under consecutive microwave irradiation using *p*-benzoquinone as the cross-linking agent. The optimum concentration of the crosslinker *p*-benzoquinone was observed to be 1.0 mM, while the relative activity of CoLG reached up to 140%, compared with that of free LEK. A partial inactivation was observed for co-immobilized LG at a higher concentration of *p*-benzoquinone (2.5 mM), due to inactivation effect of redundant *p*-benzoquinone (Fig 2).

In general, co-immobilizing two or more enzymes on the same support does not guarantee an effective process, because one immobilization chemistry might be optimal for one enzyme but deactivate the other. Therefore the co-immobilization technique must be carefully chosen to preserve activity and, ideally, to increase the stability of all of the enzymes that are involved in the biocatalytic cascade. Recently, the argrose surface that is activated by different reactive groups has been developed [5]. One of these groups is highly reactive under alkaline conditions, thereby promoting in tense covalent protein–support attachments, whereas the other group reversibly immobilizes the protein under mild pH conditions (pH 6–8). However, mass-transfer limitations may impede uniform distribution of enzymes with larger molecular size.

In this case, more of the critical residues in the catalytic center of enzymes could be crosslinked to *p*-benzoquinone with increasing concentration shown in Fig 2, which might be the major reason of inactivation effect.

### Effect of microwave power and time

**Fig 3:**
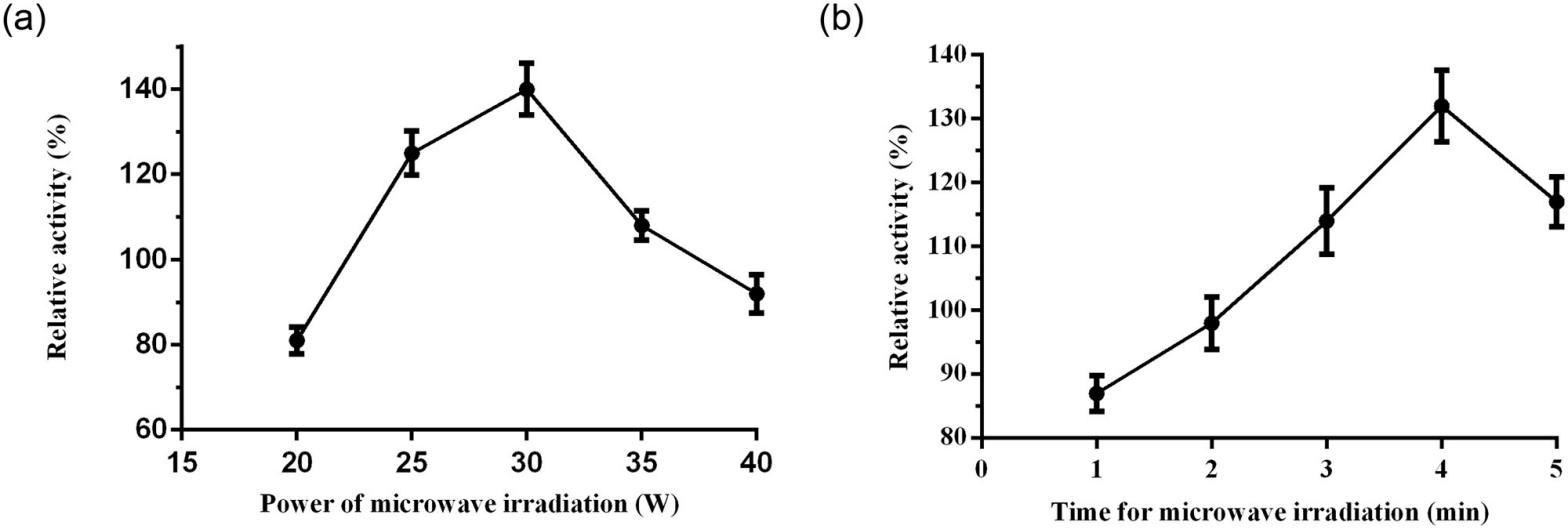
Effect of the microwave irradiation conditions on the relative activity of the covalently immobilized LEK and GDH. (a) microwave power, the set time for irradiation was 3 min. (b) irradiation time, the set power for irradiation was 30 W.

The effect of microwave irradiation power on the activity of co-immobilized LEK-GDH was observed in the power range of 20 to 40 W. The results showed that the optimal power of the microwave irradiation for the co-immobilized LG was at 30W (Fig 3a). After 4 min of microwave irradiation at 30W for the covalent immobilization, the activity of CoLG in the MCFs was 140% comparing with that of the free LEK (Fig 3b).

However, in the immobilization process, especially with the mesoporous materials, the enzyme molecules could not be well dispersed in the mesoporous carrier due to the limitation of diffusion, and often only stay in the outer channel especially for macromolecular enzymes. Appropriate microwave irradiation can modulate the structure of the enzyme and enhance the kinetics of protein folding, due to the induced bipolar molecular vibration. A higher-power or longer-term irradiation may generate excessive heat, leading to the damage of protein structure and the deceasing of the catalytic activity of enzymes. In contrast, low-power or short-term irradiation may work insufficiently. Therefore, the structure and activity of immobilized enzymes were partially influenced by microwave irradiation [19, 20].

In our previous work, the immobilization process of thermolysin with MCFs was over 20 hours, while it could be 4 minutes under microwave irradiation [21]. There are two main explanations for the shorten process. Microwave accelerates the reaction rate possibly by overheating the solvent, this leads to an increase in pressure; it may also be because microwave heating will cause some substances (such as catalysts) to generate "hot spots" higher than the surrounding temperature, resulting in faster rate. Another explanation is: (1) microwave can excite the reactant molecules, shorten the induction period of the reaction; (2) the orientation effect of the microwave field on the molecular movement, improve the effective collision frequency and speed up the reaction rate; (3) the electromagnetic field can increase the pre exponential factor and the activation energy of the reaction at the same time. However, both explanation need more proof.

### Influence of SA and CaCl2 concentration

**Fig 4:**
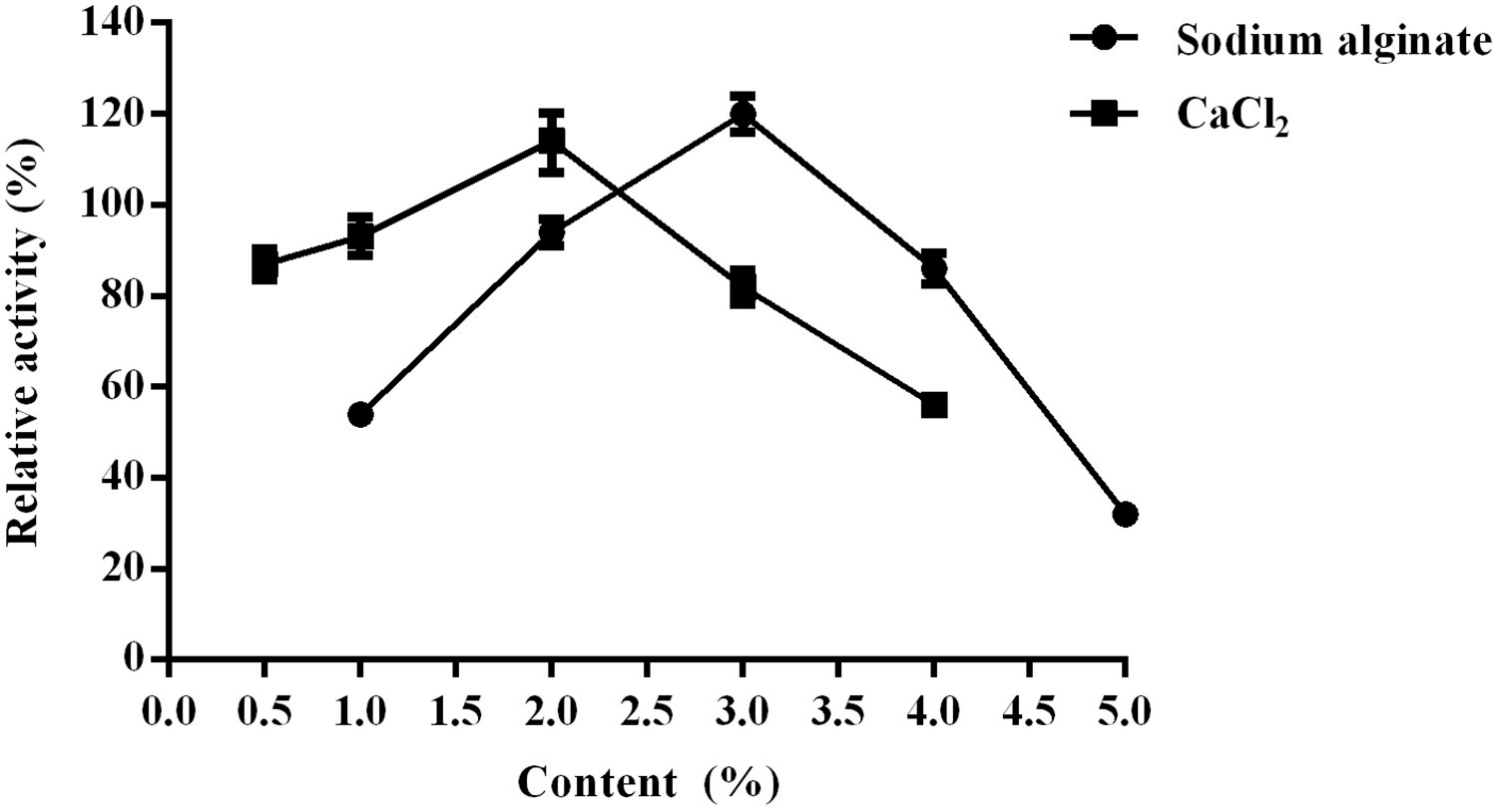
Effect of sodium alginate and CaCl_2_ concentration on the CoLG-CA preparations.

It is a mild and inexpensive method to entrap enzymes in calcium alginate (CA) gel capsules, that offers the advantage of simplicity and non-toxic character [22]. As shown in Fig 4, the relative activity of CoLG-CA was the highest, reaching at more than 100%, when the concentration of sodium alginate was 3% and that of CaCl_2_ was 2%. When the concentration of sodium alginate were lower than 3%, the forming gel particles with poor mechanical strength were too soft, leading to serious enzyme leakage. On the contrary, the increasing concentration of sodium alginate may result in the thick coating of gel capsules, leading to the difficulty of substrate-diffusion. The characteristics of enzymes immobilized in calcium alginate gel capsules, such as thickness and permeability to substrate, can be improved by changing sodium alginate and CaCl_2_ concentrations [23]. Also, the concentrate of CaCl_2_ determined the mechanical strength and solidity of gel capsules. Low concentration of CaCl_2_ may result in low crosslinking degree, incomplete encapsulation and unstable immobilized enzyme, while pore sizes of gel particles were too small to form tight intermolecular crosslinking under high concentrate of CaCl_2_ [24].

**Fig 5:**
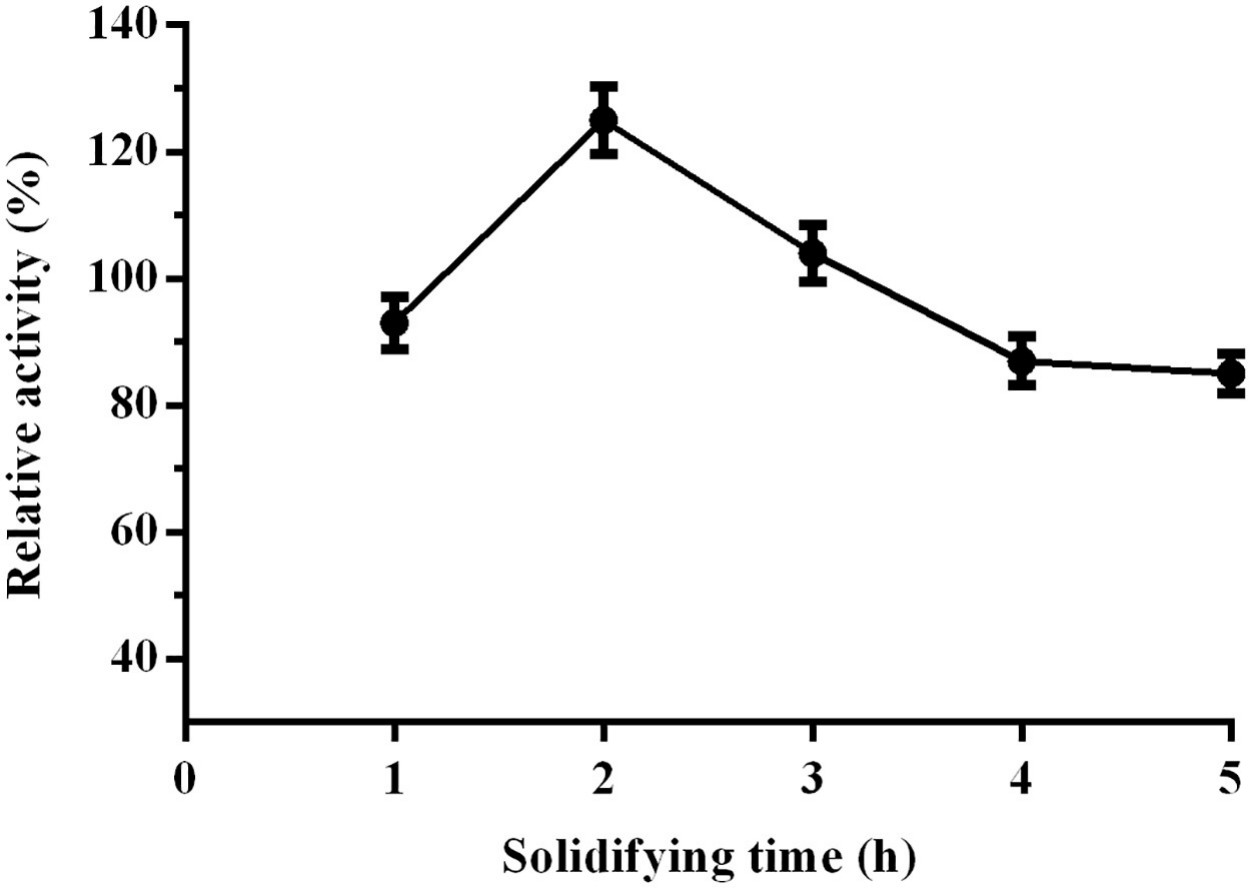
Influence of the solidification time in 2.0% CaCl_2_ solution on CoLG-CA preparations.

The mixed solution of enzymes and sodium alginate reacted with 2% CaCl_2_ for 2-hour solidifying, the resulting CoLG showed the highest relative activity, reached at 1.25 times activity of that of free LEK. Sodium in sodium alginate are replaced by Ca^2+^ to form calcium alginate, and the reaction would affect by the solidifying time, resulting in forming the stable structure of gel capsules (Fig 5).

To sum up, it is a better immobilization strategy to link enzymes and carriers by covalent combination. The advantage of this method is that the covalent bond show relatively strong binding force, so the enzyme will not be easily removed from the surface of the carriers. Moreover, once the enzyme is permanently inactivated, the carrier can be used again.

### Temperature effect

**Fig 6:**
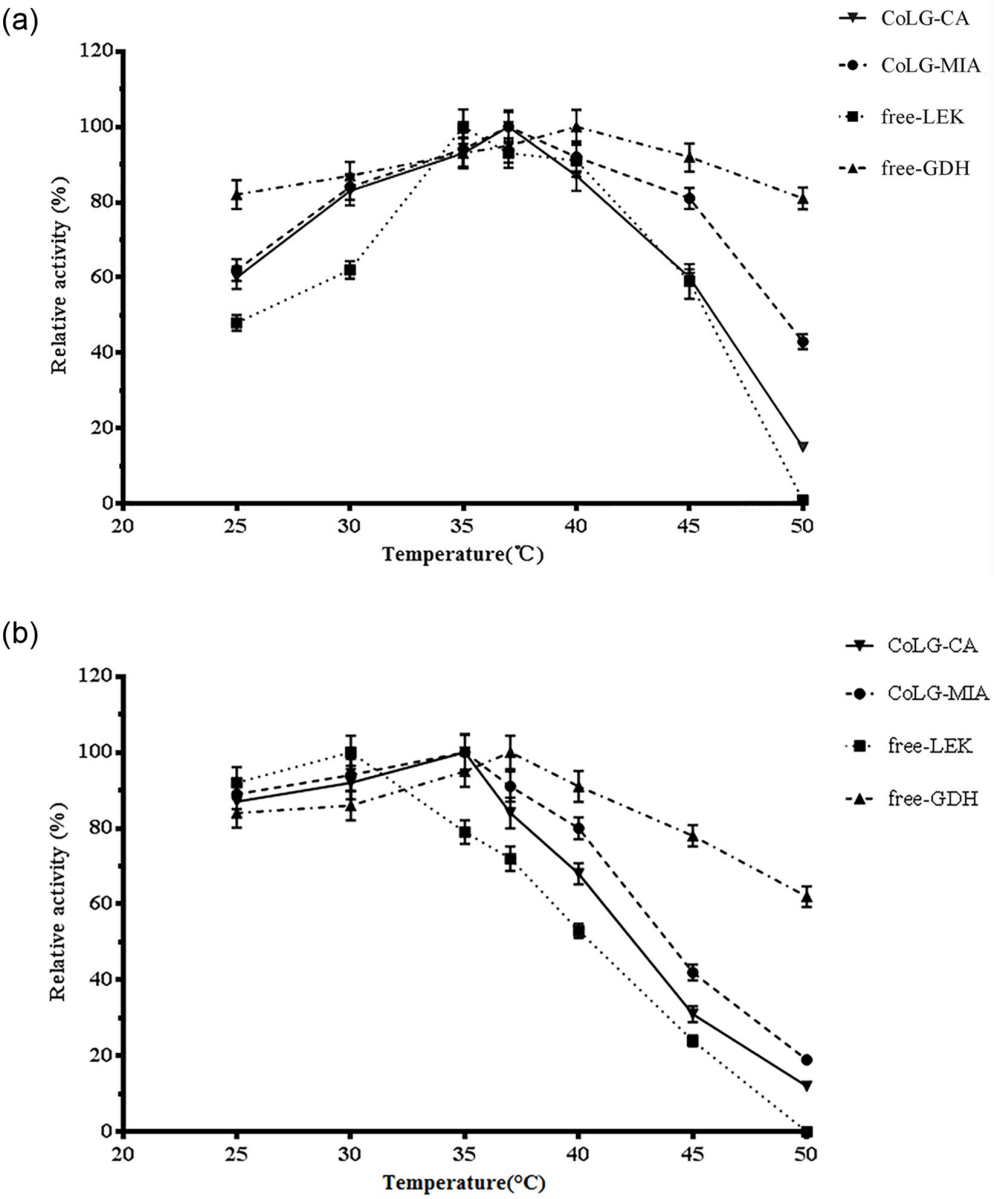
Optimum reaction temperature (a) and stability of temperature (b) for co-immobilized preparations and free enzymes.

The effects of temperature on the activities of LEK, GDH and co-immobilized LEK-GDH preparations were observed in the range of 25 to 50°C. As shown in Fig 6, the optimal temperatures of LEK, GDH, CoLG-MIA, CoLG-CA were 35, 40, 37 and 37°C, respectively (Fig 6a). The free GDH exhibited relative higher residual activities from 25 to 50°C, while the free LEK lost more than 40% activities under 30°C or over 45°C. However, the CoLG-MIA exhibited a medium influence by temperature, compared with CoLG-CA (Fig 6b). This meant that the CoLG-MIA would work at 37°C with increased operational stability and simplified operational process.

It has been reported that multi-point immobilization can significantly improve the thermal stability of the immobilized enzyme. In general, the conformation of the enzyme with multi-binding sites is more stable. However, the redundant active groups on the surface of the carrier may also have some subsequent adverse reactions with the enzyme, that may increase the possibility of enzyme deactivation.

Uniform distribution of two enzymes in the MCFs support by the microwave irradiation-assisted method improved their thermal stabilities. In previous study, microwave irradiation-assisted method allowed the entire reaction medium to be heated uniformly, thus speeded up the enzymatic reactions, reduced the inactivation of enzyme under the operational conditions, such as higher temperature or high substrate concentration [25]. Moreover, an additional mass transport driving force by microwave irradiation would accelerate the diffusion and inhibit the autolysis of enzyme. Therefore, it is reasonable to infer that the microwave irradiation had a positive effect on the enzyme stability.

In contrast, the mechanical strength of CoLG-CA seriously decreased with the rise of temperature, leading to enzymes`leakage and inactivation. Moreover, immobilization by entrapment showed some disadvantages such as diffusional limitation and delayed attainment of equilibrium [14]. Especially, if the catalytic reaction took place rapidly, the accumulation of reactive products would be quickly formatted, making it difficult to release from capsules. That may lead to a decline in reaction rate and the rupture of capsules [26].

### pH effect

**Fig 7:**
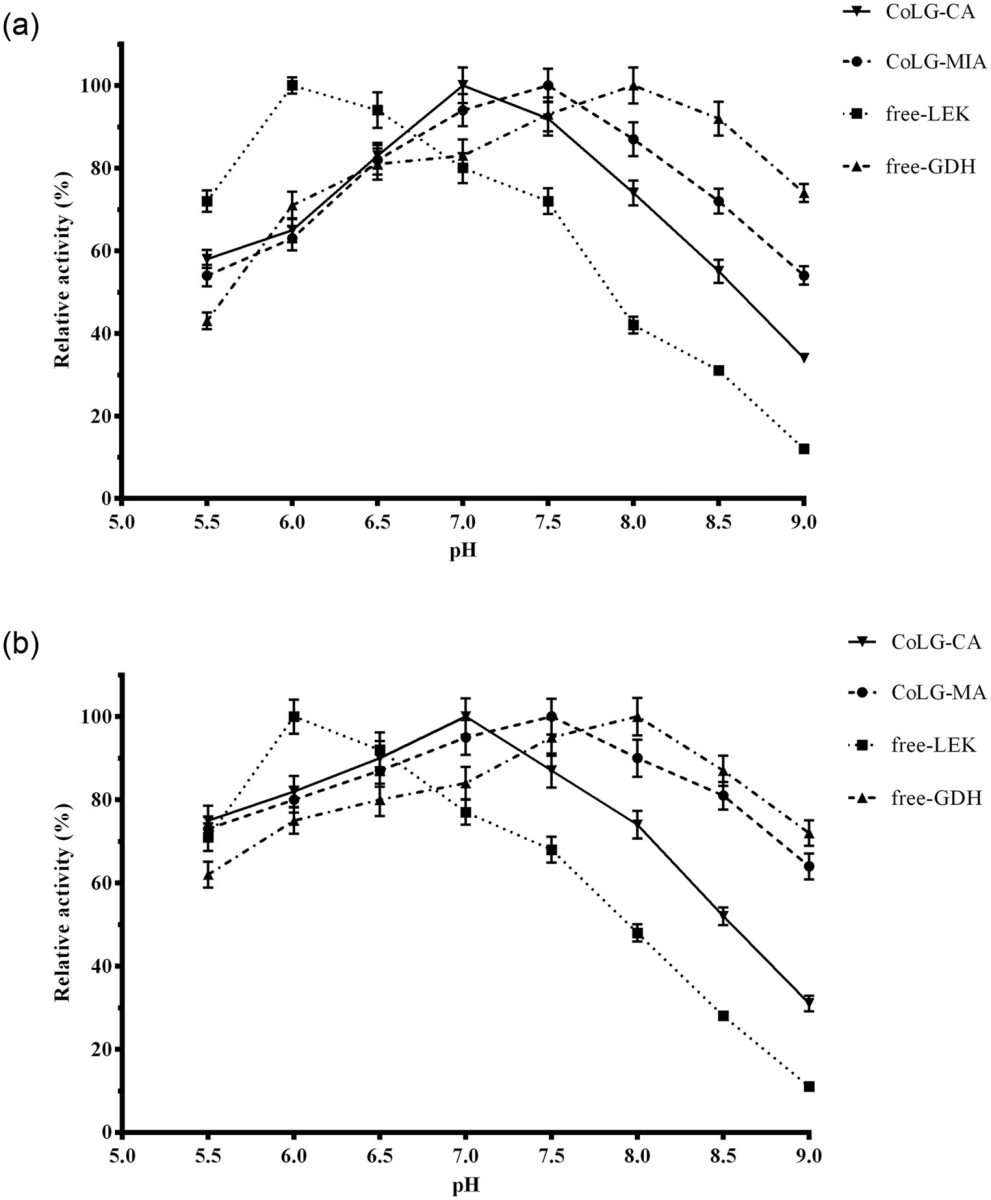
Optimum reaction pH (a) and pH stability (b) for co-immobilized preparations and free enzymes.

The optimum pH of LEK, GDH, CoLG-MIA, CoLG-CA were 6.0, 8.0, 7.0 and 7.5, respectively (Fig 7a). Compared with the free LEK, both of CoLG were more stable from pH 7.0 to pH 9.0 during 60 min incubation. Also, both of co-immobilized LEK-GDH preparations obtained the improved stabilities in the optimum reactive buffer. Moreover, the CoLG-MIA showed more stabilities in alkaline buffers than CoLG-CA (Fig 7b). This may result from the fact that the C-O and C-N bonds were stable in acidic conditions [27]. As previously reported, the immobilized enzymes by microwave-assistance method also showed better stabilities in the acidic and alkaline conditions [23].

### Storage and batch operational stability

**Fig 8:**
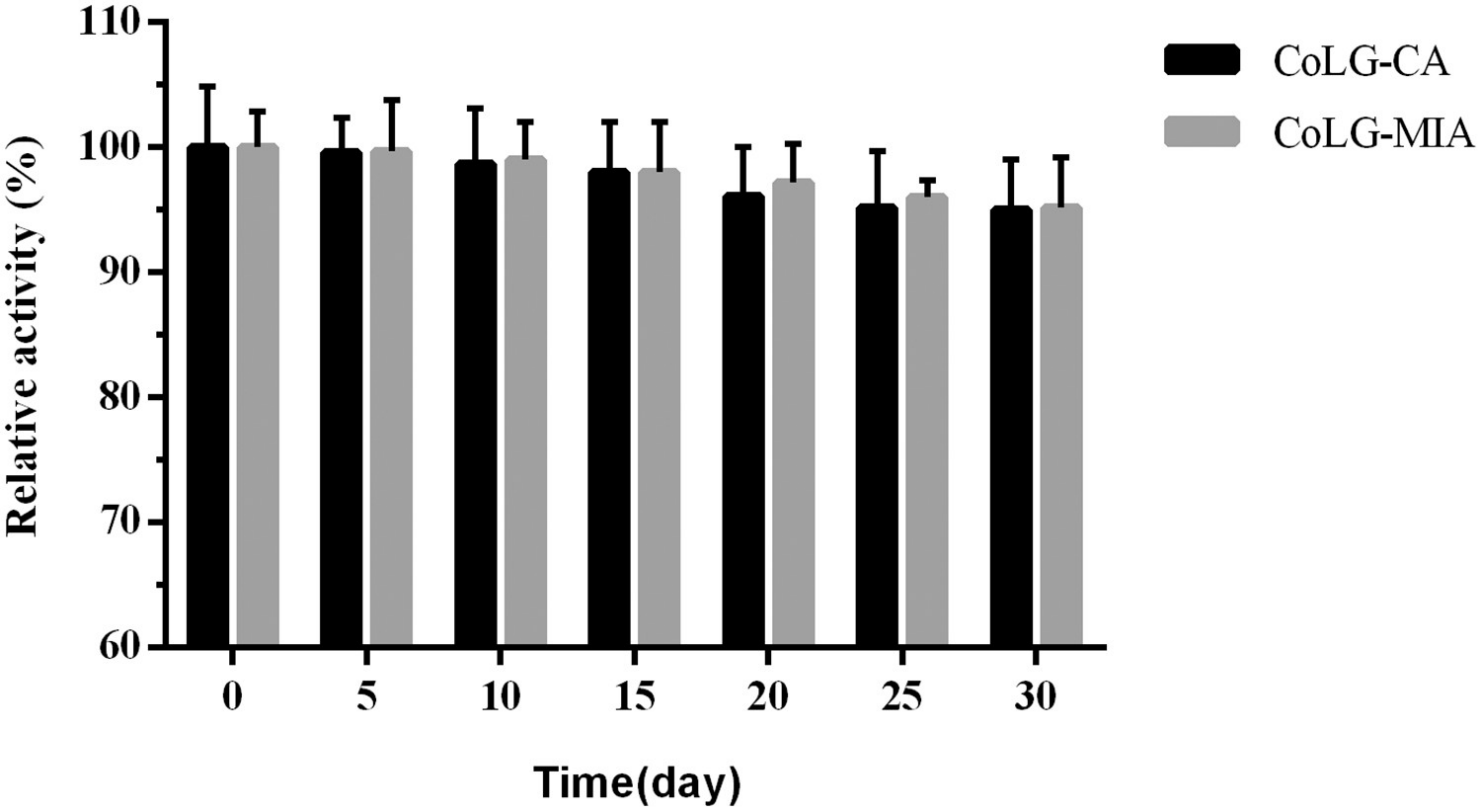
Storage stability of two immobilized LEK and GDH preparations.

To further analyze the advantage of covalent immobilization of LEK and GDH, the storage stabilities of the immobilized LEK-GDH preparations were investigated. No loss of activity was observed over a week. More than 90% of residual activity was maintained after storage for 30 days. No obvious difference was observed between two CoLG preparations (Fig 8). Therefore, co-immobilized LEK and GDH preparations can be stored at 4°C stably for 30 days.

**Fig 9:**
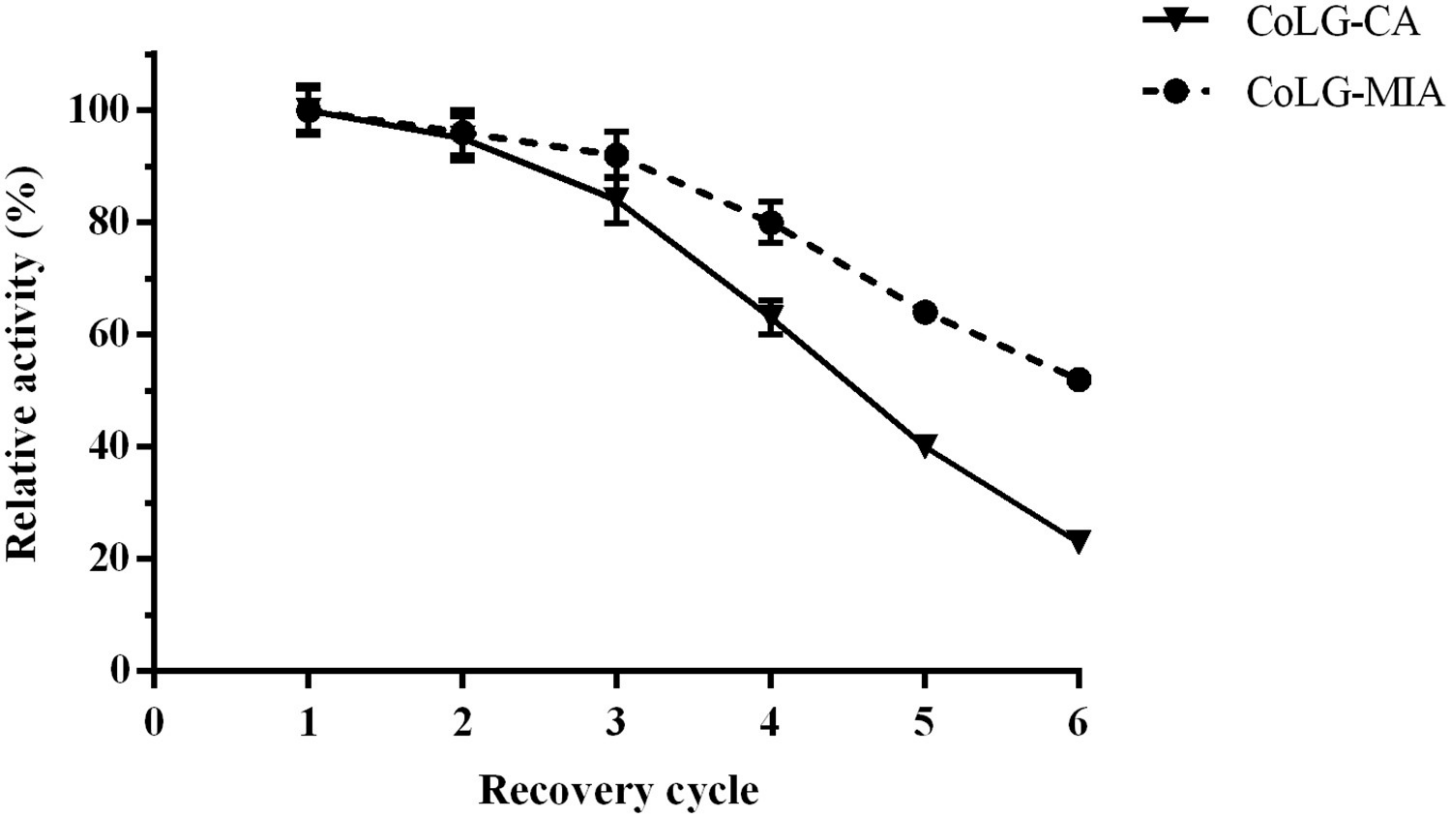
Batch operational stability of two immobilized LEK and GDH preparations.

Immobilized enzyme can be easily separated from the reactive mixture and recycled multiple times, which provides an economic and convenient alternative as catalyst [24]. The residual activity was measured after repeated cycles of the recovery process. The residual relative activity of CoLG-MIA maintained at approximately 50% after six cycles of the repeated recovery process. Meanwhile, that of CoLG-CA was 23% under the same operation (Fig 9). Microwave irradiation could distribute uniformly enzymes in the reaction medium and enhance covalent binding of aminated MCFs with enzymes, resulting in high stability in batch operation [13]. In contrast, enzymes embedded in calcium alginate gel capsules would easily drop from carrier, leading to enzyme loss.

## Conclusions

In this study, an efficient and stable bi-enzymatic cascades for bio-redox system was established by covalent co-immobilization with consecutive microwave irradiation assistance. When the output power was 30 W for 4 min, LEK and GDH were co-immobilized on MCFs cross-linked with 1.0 mM p-benzoquinone. All enzymes were immobilized on carriers without being detected in the suspension after immobilization. The relative activity of the immobilized enzyme (CoLG-MIA) was 140% comparing with that of free LEK. When 3% sodium alginate and 2% CaCl_2_ were used, LEK and GDH were embedded, yielding the CoLG-CA with 1.25 times activity of the free LEK. Both immobilized enzymes exhibited enhanced thermal, storage, and operational stabilities, compared with free LEK. The increasing of activity and stability was due to many reasons, such as enzyme conformation changes after interactions with the support, substrates partition, coenzyme partition, etc., however, the contribution of shorter immobilization time and enhanced spatial distribution under the microwave irradiation might be important to obtain the immobilized catalyst. As a result, the CoLG-MIA showed the highest thermal stability at optimal temperature. The stable and efficient immobilization technique with MIA could also be applied to other enzymes to pursue an ideal biocatalyst.

## Supporting information

S1 Raw image (PDF)
