# Supplementary material for: Stable and efficient immobilization of bi-enzymatic NADPH cofactor recycling system under consecutive microwave irradiation
Source: PLoS One. 2020 Nov 18;15(11):e0242564. doi: 10.1371/journal.pone.0242564 (PMC7673530; doi:10.1371/journal.pone.0242564)

Fig 1 Expression and purification of His-tagged proteins. (a) LEK, (b) GDH. M: protein molecular weight maker, Lane 1: the whole cell lysates before induction, Lane 2: the whole cell lysates after IPTG induction, Lane 3: soluble fraction of the whole cell lysates after IPTG induction, Lane 4–6: eluates collected with 100, 200 and 500 mM imidazole, respectively.

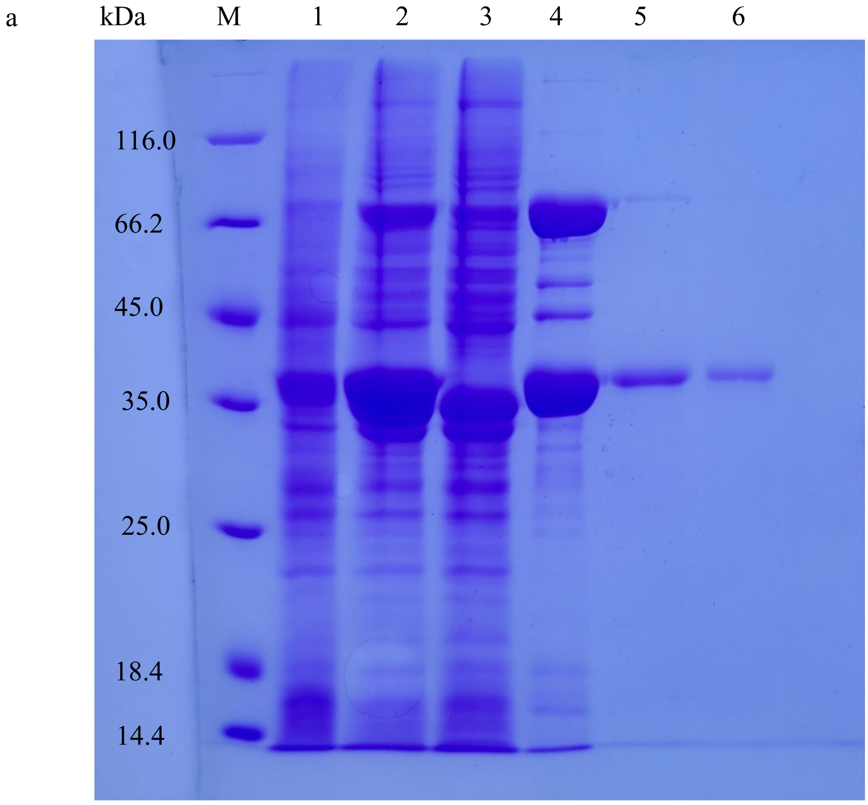
a
kDa
M
1
2
3
4
5
6
116.0
66.2
45.0
35.0
25.0
18.4
14.4

b

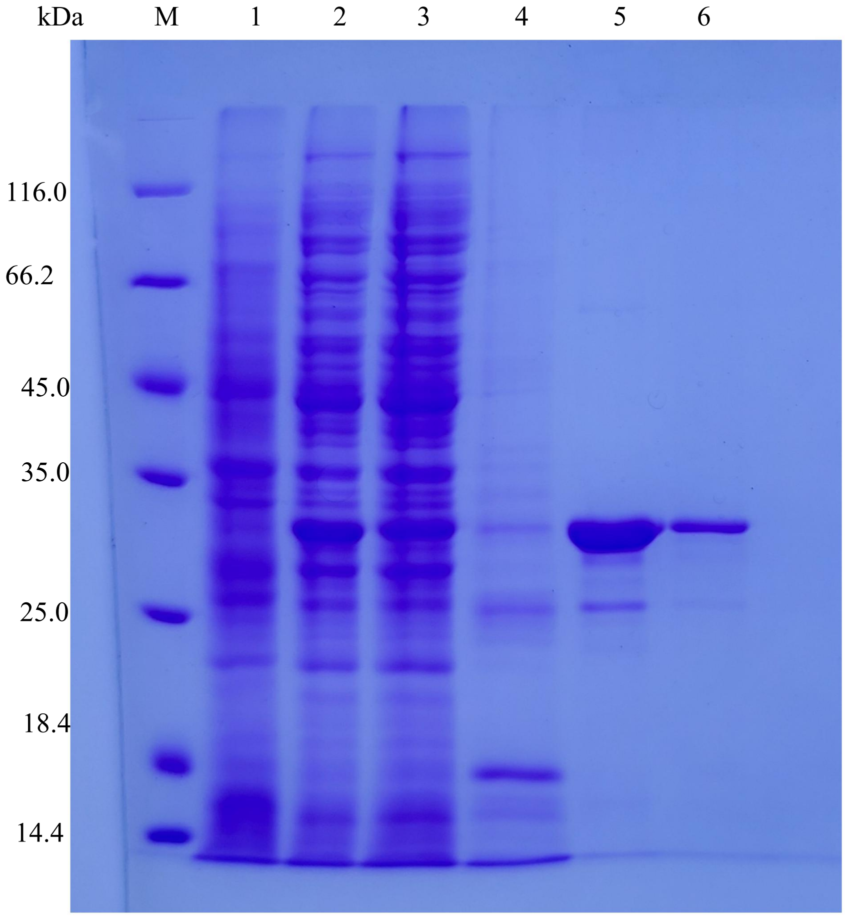

Supplement: S1 Raw image — (PDF) [file pone.0242564.s001.pdf]
